# Acute Amiodarone Pulmonary Toxicity after Drug Holiday: A Case Report and Review of the Literature

**DOI:** 10.1155/2015/927438

**Published:** 2015-05-14

**Authors:** Ahmed Abuzaid, Marwan Saad, Mohamed Ayan, Amjad Kabach, Toufik Mahfood Haddad, Aiman Smer, Amy Arouni

**Affiliations:** ^1^Department of Internal Medicine, Creighton University, Omaha, NE 68131, USA; ^2^Department of Internal Medicine, Seton Hall University School of Health and Medical Sciences, Trinitas Regional Medical Center, Elizabeth, NJ 07207, USA; ^3^Department of Cardiology, Ain Shams University, Cairo 11381, Egypt; ^4^Department of Cardiology, Creighton University, Omaha, NE 68131, USA

## Abstract

Amiodarone is reported to cause a wide continuum of serious clinical effects. It is often challenging to detect Amiodarone-induced pulmonary toxicity (AIPT). Typically, the diagnosis is made based on the clinical settings and may be supported by histopathology results, if available. We describe a 57-year-old patient who developed severe rapidly progressive respiratory failure secondary to AIPT with acute bilateral infiltrates and nodular opacities on chest imaging. Interestingly, Amiodarone was discontinued 3 weeks prior to his presentation. He had normal cardiac filling pressures confirmed by echocardiography. To our knowledge, this is the first case of isolated acute lung injury induced by Amiodarone, three weeks after therapy cessation, with adequate clinical improvement after supportive management and high dose steroid therapy.

## 1. Introduction

As Amiodarone is a commonly prescribed medication, there should be a high clinical suspicion for the development of Amiodarone-induced pulmonary toxicity (AIPT), allowing for timely detection to prevent any unfavorable outcomes. AIPT should be on our list of differential diagnosis for any patient suffering acute onset deterioration of respiratory function with current or recent use of Amiodarone.

## 2. Case Presentation

A 57-year-old Caucasian male with past medical history of hypertension and sick sinus syndrome had undergone successful pacemaker insertion 8 months ago. One month later, he developed recurrent bouts of atrial fibrillation and was started on Amiodarone 400 mg daily, as well as Metoprolol and oral anticoagulation. He maintained sinus rhythm for 6 months, so Amiodarone was interrupted. Three weeks after discontinuation of Amiodarone, he was evaluated in the emergency department because of right lateral sharp chest pain and worsening shortness of breath that started 2 days prior to his presentation. He had no fever, chills, weight loss, palpitations, or leg swelling. He had a remote tobacco history. Chest X-ray during his last admission was unremarkable.

On physical examination, the patient appeared ill and tachypneic with respiratory rate of 28 breaths per minute. He was afebrile, with a blood pressure of 115/74 mmHg and heart rate of 60 bpm. His oxygen saturation was 86% on room air. Cardiac examination showed normal heart sounds with no audible murmurs or gallops. Chest auscultation revealed diminished breath sounds mainly at the lung bases bilaterally. His abdomen was soft. His legs were well perfused, with no edema or calf tenderness. No skin rash, clubbing, or enlarged lymphadenopathy was detected.

**Figure 1:**
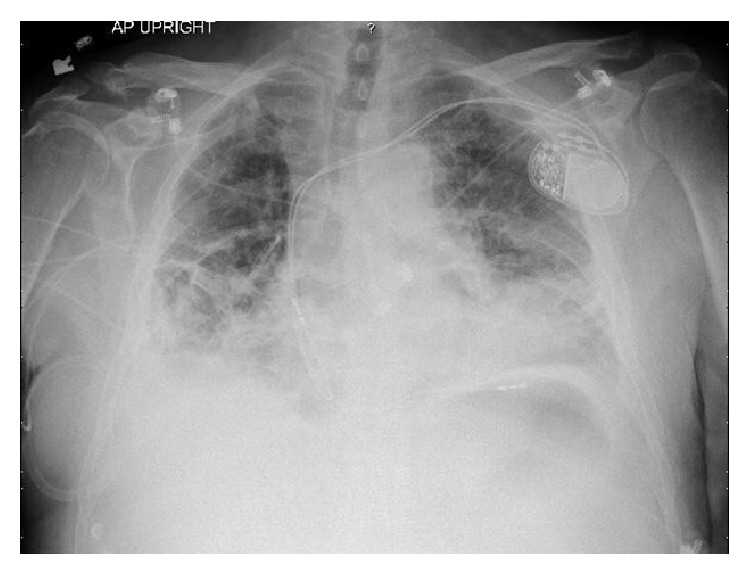
Chest X-ray on presentation showing bilateral lower lobe consolidations.

**Figure 2:**
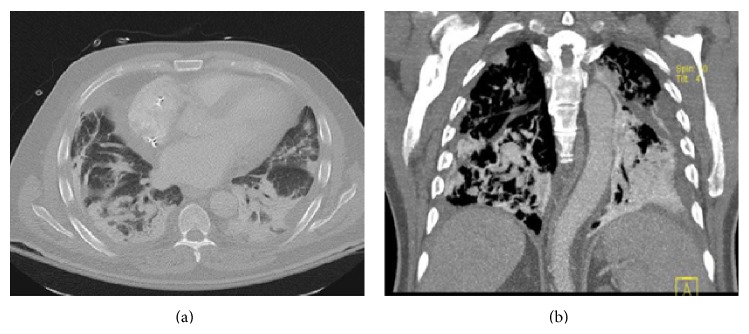
(a) and (b) CT chest with contrast showing high attenuation areas of multiple conglomerate consolidation in both lung bases ((a) axial cut; (b) coronal cut).

Laboratory results revealed leukocytosis with neutrophilia and elevated inflammatory markers. International Normalized Ratio (INR) was 3.1 and cardiac peptide was unremarkable. Arterial blood gas showed significant hypoxemia. Chest X-ray revealed bilateral lower lobe consolidations and stable pacer lead position (Figure 1). An electrocardiogram (EKG) showed a paced rhythm with first degree AV block and prolonged QT interval. CT chest with contrast was noteworthy for focal high attenuation areas of multiple conglomerate consolidation in both lung bases with no evidence of pulmonary embolism (Figures 2(a) and 2(b)). Echocardiography was unremarkable.

**Figure 3:**
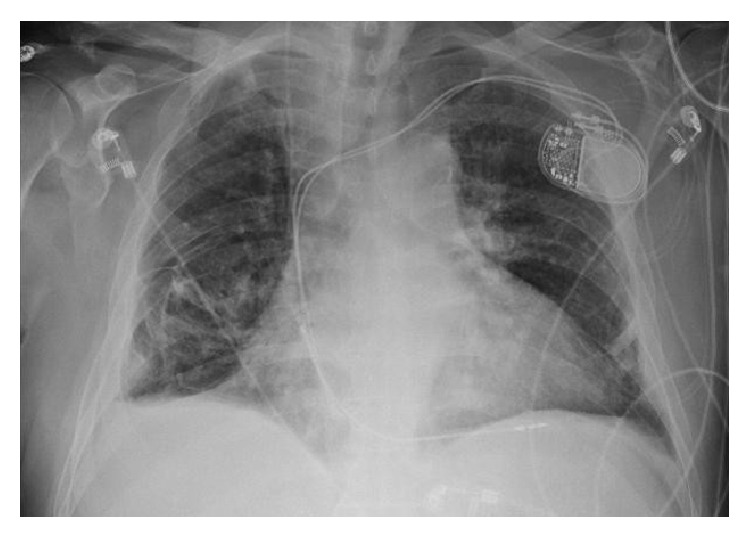
Follow-up chest X-ray showing improvement of lung consolidations.

Given his acute presentation, bilateral pulmonary disease, and absence of left heart failure, supportive therapy was started together with antibiotics for possible pneumonia in addition to high dose systemic corticosteroids. Amiodarone level was 3.2 mcg/mL (reference range: 0.5–2.0 mcg/mL). Patient refused bronchoscopy and lung biopsy. Extensive workup ruled out the possibility of an infectious process, rheumatologic or granulomatous diseases. PET scan omitted underlying lung malignancy. HIV test was nonreactive. The patient exhibited significant clinical and radiological improvement with decline of his oxygen requirements during his hospital stay and was discharged after 5 days on steroid therapy. Chest X-ray done after 4 weeks showed a significant resolution of lung consolidations (Figure 3). Although we do not have a definite tissue diagnosis, we believe that the reason behind his acute respiratory deterioration was AIPT.

## 3. Discussion

Amiodarone became widely accepted for managing a broad spectrum of tachyarrhythmias [1]. Imminent side effects as skin photosensitivity, corneal deposits, thyroid dysfunction, liver dysfunction, lung injury, coagulopathy, and neuropathy were described in numerous reports [1, 2]. However, of all, pulmonary toxicity is the most serious [1]. Fatal complications of Amiodarone include adult respiratory distress syndrome (ARDS) with mortality rate of 50% [3], advanced pulmonary fibrosis [4], and malignant dysrhythmias. The incidence of AIPT varies between 0.5 and 17% on long-term use [2, 3] and is considered a difficult clinical challenge by most available diagnostic tools.

Despite being given to target the heart, Amiodarone usually has a higher pulmonary than cardiac concentration [5]. It has a long half-life of 30–108 days [4]. Desethylamiodarone, the active metabolite, penetrates lung tissues 5 times more than the parent drug and provides a depot release with unpredictable serum levels [5].

AIPT is a multifactorial process. Suggested direct pulmonary toxicity includes reduced phospholipid degradation and, hereby, leads to its accumulation [4, 6, 7], and, consequently, lipid-laden macrophages formation, lipid peroxidation, reactive oxygen radicles generation, disturbance of cellular calcium and prostaglandin metabolism, and deposition of collagens resulting in lung injury [2, 4, 8]. Eventually, interstitial inflammation follows [2, 7, 9], mimicking infectious, granulomatous, or neoplastic diseases [3, 10]. Of note, the asymptomatic lipoid pneumonia is a unique drug effect rather than toxicity [10].

Amiodarone dosages are independent determinants for lung toxicity. Therapy for 6–12 months or more represents the highest risk during treatment and/or even after discontinuation [3, 4], as in our patient. Those with cumulative dose ≥100 g or ≥400 mg/day for more than two months, or 200 mg/day for two years, form a high risk group [2, 3]. Not often, lower doses (≤200 mg/d) have also been reported [2, 3]. Other risk factors are male gender, previous lung disease, history of cardiothoracic surgery, high FiO2 levels, and iodinated contrast [2–4, 8, 9]. Our patient had some of these high risk criteria.

AIPT may develop within the first few days of treatment and up to several years later. Progressive exertional dyspnea has been widely reported [1]. Low-grade fever and weight loss have been described. Dry cough is common; sputum production appears to be unusual where hemoptysis is rare [1]. These symptoms can be masked by preexisting pulmonary disorder or overt cardiac failure [4]. Severe cases can exhibit diffuse rales and hypoxemia [2, 4].

There is no evidence to attest Amiodarone assays as a confirmatory tool for drug toxicity [7]. Early radiographic features include localized or diffuse interstitial infiltrates or alveolar ground-glass shadows, universally in the right lobe [2, 3]. Pleural thickening and/or effusion are uncommon and upper lobe masses can be noted [2]. CT scans offer a unique ability to assess increased lung density yet cannot exclude the normal drug accumulation in lung tissues [8]. Lung scintigraphy, although nonspecific [5], is still useful to distinguish any associated heart failure [2, 7].

PFTs typically reveal a restrictive pattern with a decreased diffusion lung capacity (DLCO) of 15–20%, as an early disease indicator [1]. As CHF is prevalent in this population, minor changes in DLCO would have low specificity in the detection of Amiodarone toxicity [3]. A tissue biopsy is suggested if symptoms do not recover within 1 or 2 months to rule out other diagnostic enigmas as infection, pulmonary fibrosis, and malignancy [4].

At present, the mainstay of therapy is drug discontinuation [1, 2]. Systemic corticosteroids had been widely accepted, despite the lack of well-controlled studies, even in the absence of hypoxemia [4]. Due to its prolonged metabolism, pulmonary toxicity may initially progress despite drug discontinuation and may recur after steroid withdrawal [2, 4]. Mechanical ventilation may be required in severe cases [10]. Corticosteroids are suggested for at least 4–12 months to avoid relapse [4]. Radiologic resolution can take up to 18 months [3, 4].

## 4. Conclusion

What clinical features suggest a diagnosis of Amiodarone-induced lung injury? A high index of suspicion must be retained for pulmonary toxicity in patients receiving or who have received Amiodarone, particularly in those with high risk features. If development of new respiratory illness and radiological findings without other recognized causes exists, then Amiodarone toxicity should be presumed. Diagnosis is often done by exclusion as there is no specific confirmatory clinical or diagnostic test. Early supportive care and corticosteroids should be employed as a practical treatment scheme.
