# Corrigendum to “Acute Amiodarone Pulmonary Toxicity after Drug Holiday: A Case Report and Review of the Literature”

**DOI:** 10.1155/2015/182154

**Published:** 2015-10-19

**Authors:** Ahmed Abuzaid, Marwan Saad, Mohamed Ayan, Amjad Kabach, Toufik Mahfood Haddad, Aiman Smer, Amy Arouni

**Affiliations:** ^1^Department of Internal Medicine, Creighton University, Omaha, NE 68131, USA; ^2^Department of Internal Medicine, Seton Hall University School of Health and Medical Sciences, Trinitas Regional Medical Center, Elizabeth, NJ 07207, USA; ^3^Department of Cardiology, Ain Shams University, Cairo 11381, Egypt; ^4^Department of Cardiology, Creighton University, Omaha, NE 68131, USA

In the paper titled “Acute Amiodarone Pulmonary Toxicity after Drug Holiday: A Case Report and Review of the Literature” [1], the fifth author's name (Toufik Mahfood Haddad) is corrected as follows. First name is Toufik and last name is Mahfood Haddad.
